# Functional Properties and Molecular Architecture of Leukotriene A4 Hydrolase, a Pivotal Catalyst of Chemotactic Leukotriene Formation

**DOI:** 10.1100/tsw.2002.810

**Published:** 2002-06-26

**Authors:** Jesper Z. Haeggström, Pär Nordlund, Marjolein M.G.M. Thunnissen

**Affiliations:** Department of Medical Biochemistry and Biophysics, Division of Chemistry 2, Karolinska Institutet, S-171 77 Stockholm, Sweden; 2Department of Biochemistry, University of Stockholm, Arrhenius Laboratories A4, S-106 91 Stockholm, Sweden

**Keywords:** inflammation, lipid mediators, lipoxygenase, leukotrienes, leukotriene B_4_, leukotriene A_4_, epoxide hydrolase, aminopeptidase, zinc, crystal structure, antiinflammatory drugs, structure-based drug design

## Abstract

The leukotrienes are a family of lipid mediators involved in inflammation and allergy. Leukotriene B_4_ is a classical chemoattractant, which triggers adherence and aggregation of leukocytes to the endothelium at only nM concentrations. In addition, leukotriene B_4_ modulates immune responses, participates in the host defense against infections, and is a key mediator of PAF-induced lethal shock. Because of these powerful biological effects, leukotriene B4 is implicated in a variety of acute and chronic inflammatory diseases, e.g., nephritis, arthritis, dermatitis, and chronic obstructive pulmonary disease. The final step in the biosynthesis of leukotriene B_4_ is catalyzed by leukotriene A_4_ hydrolase, a unique bifunctional zinc metalloenzyme with an anion-dependent aminopeptidase activity. Here we describe the most recent developments regarding our understanding of the function and molecular architecture of leukotriene A_4_ hydrolase.

## INTRODUCTION

The development and maintenance of inflammation are governed by a complex network of cellular and humoral factors. Among these are the eicosanoids, a class of structurally related paracrine hormones derived from the oxidative metabolism of arachidonic acid that includes the prostaglandins, the leukotrienes, and the lipoxins[1].

The leukotrienes (LTs) are a family of eicosanoids that function as potent chemical mediators in a variety of allergic and inflammatory reactions[2,3]. In the biosynthesis of LTs, 5-lipoxygenase converts arachidonic acid, released from membrane phospholipids by cytosolic phospholipase $A_2$ ($cPLA_2$), into the unstable epoxide $LTA_4$ (Fig. 1). This intermediate may in turn be conjugated with GSH to form $LTC_4$, the parent compound of the spasmogenic cysteinyl-containing leukotrienes (cys-LT = $LTC_4$, $LTD_4$, $LTE_4$), or hydrolyzed into the proinflammatory lipid mediator $LTB_4$, in a reaction catalyzed by $LTA_4$ hydrolase. The enzyme product $LTB_4$ is a classical chemoattractant and triggers adherence and aggregation of leukocytes to the endothelium at nM concentrations[4]. In addition, $LTB_4$ modulates immune responses[5], participates in the host defense against infections[6,7], and is a key mediator of PAF-induced lethal shock[8,9]. These effects are signaled via a specific, high-affinity, G-protein coupled receptor for $LTB_4$ *($BLT_1$)*[10]. In addition, a second receptor for $LTB_4$ *($BLT_2$)* was recently discovered, the functional role of which is presently not known[11]. Interestingly, $LTB_4$ is also a natural ligand of the PPARα class of nuclear receptors and it has been suggested that it plays a role in lipid homeostasis[12]. This article describes functional and molecular properties of $LTA_4$ hydrolase/aminopeptidase, a unique zinc metalloenzyme that catalyzes the final and committed step in $LTB_4$ biosynthesis.

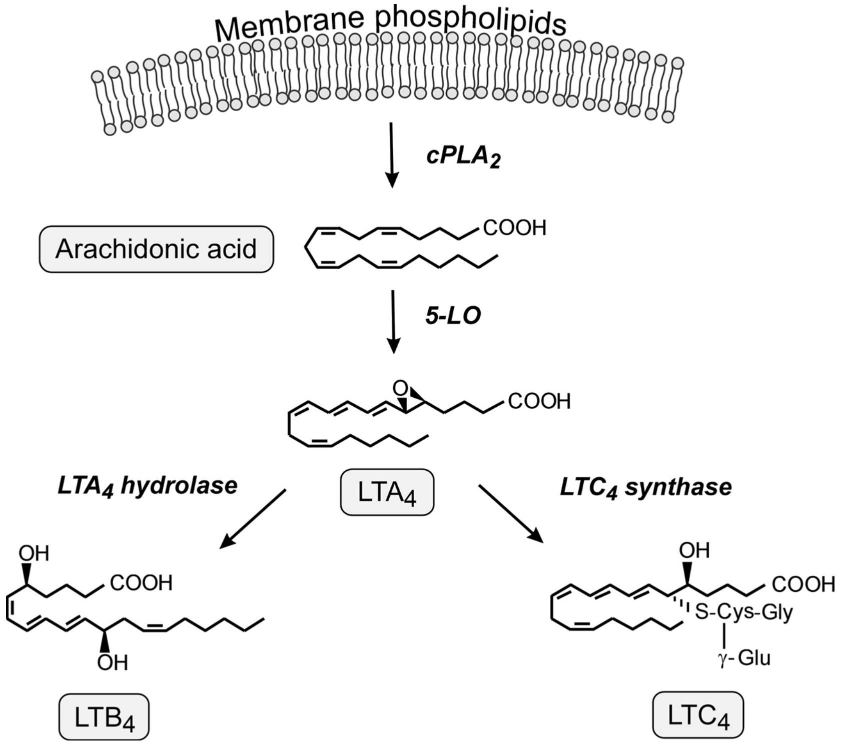

**FIGURE 1.** Biosynthesis of leukotrienes. Arachidonic acid is liberated from membrane phospholipids by cytosolic phospholipase $A_2$ ($cPLA_2$). The fatty acid is transformed by 5-lipoxygenase (5-LO) into the unstable epoxide $LTA_4$. This transient intermediate is the substrate for $LTA_4$ hydrolase and $LTC_4$ synthase to produce $LTB_4$ and $LTC_4$, respectively.

## LEUKOTRIENE $A_4$ HYDROLASE, A ZINC-DEPENDENT EPOXIDE HYDROLASE AND AMINOPEPTIDASE

Leukotriene $A_4$ hydrolase has been purified from several mammalian sources, and cDNAs encoding the human, mouse, rat, and guinea pig enzymes have been cloned and sequenced[13]. The primary structure of $LTA_4$ hydrolase harbors a typical zinc binding site with the signature HEXXH-$(X)_{18}$-E[14,15] containing one zinc atom, the primary role of which is catalytic[16,17]. In addition to its well-characterized epoxide hydrolase activity (the conversion of $LTA_4$ into $LTB_4$), $LTA_4$ hydrolase possesses an anion-dependent peptide cleaving activity[17,18]. This aminopeptidase activity accepts a variety of substrates, and certain arginyl di- and tripeptides as well as *p*-nitroanilide derivatives of Ala and Arg are hydrolyzed with high efficiencies[19]. Although it has never been experimentally verified, it is generally assumed that the aminopeptidase activity is involved in the processing of peptides related to inflammation and host defense. Because of the strong stimulatory effects of monovalent anions, in particular chloride and albumin, an extracellular role for the peptidase activity has been discussed[20,21].

Based on its zinc signature and aminopeptidase activity, $LTA_4$ hydrolase is now classified as a member of the M1 family of zinc metallopeptidases[22]. This family includes enzymes such as aminopeptidase A (EC 3.4.11.7, APA), aminopeptidase B (EC 3.4.11.6, APB), and aminopeptidase N (EC 3.4.11.2, APN).

## SUICIDE INACTIVATION OF $LTA_4$ HYDROLASE

A characteristic feature of $LTA_4$ hydrolase is the inactivation and covalent modification by its substrate $LTA_4$ that occurs during catalysis[23,24,25]. We used differential Lys-specific peptide mapping to identify a 21-residue peptide, denoted K21, which was modified by $LTA_4$ during this process[26]. Tyr-378 was identified as the site of attachment between lipid and protein; to study the role of this residue in suicide inactivation and its potential catalytic function, we carried out a mutational analysis[26]. Interestingly, removal of the phenolic hydroxyl group of Tyr-378 yielded mutated enzymes that are protected from inactivation by $LTA_4$. Mutants in position 378 are also able to generate not only the natural $LTB_4$, but also the geometrical isomer $\Delta^6$-*trans*-$\Delta^8$-*cis*-$LTB_4$, in a yield of about 20 to 30% of that of $LTB_4$[27]. This indicates that Tyr-378 is involved in catalysis, perhaps by assisting in the proper alignment of $LTA_4$ in the substrate-binding pocket or by promoting a favorable conformation of a putative carbocation intermediate.

## IDENTIFICATION OF AMINO ACIDS INVOLVED IN METAL BINDING AND CATALYSIS

Using biochemical techniques, site-directed mutagenesis, and, recently, x-ray crystallography, a number of amino acid residues that play important functional roles have been identified (Table 1).

### The Zinc Binding Ligands

The three proposed zinc binding ligands, His-295, His-299, and Glu-318, were verified by site-directed mutagenesis followed by zinc analysis and activity determinations of the purified mutated proteins[28]. None of the mutants contained significant amounts of zinc and all were enzymatically inactive, demonstrating the critical role of the zinc for both enzyme activities.

**TABLE 1**
**Functional Residues in $LTA_4$ Hydrolase**

| Residue | Enzyme reaction | | Function |
|---|---|---|---|
| | Epoxide hydrolase | Aminopeptidase | |
| His-295 | + | + | Zinc ligand |
| His-299 | + | + | Zinc ligand |
| Glu-318 | + | + | Zinc ligand |
| Glu-296 | | + | General base catalyst |
| Tyr-383 | | + | Proton donor |
| Glu-271 | | + | N-terminal recognition site |
| | + | | Epoxide activation |
| Tyr-378 | + | | Suicide inactivation |
| | + | | Substrate alignment |

## The Catalytic Residues Glu-296 and Tyr-383

From x-ray crystallographic studies on thermolysin, a conserved glutamic acid residue located next to the first zinc binding ligand has been suggested to play a critical role in the reaction mechanism[29,30]. When the corresponding residue in $LTA_4$ hydrolase, Glu-296, was substituted for a Gln, Ala, Asp, or Asn by site-directed mutagenesis, the enzyme lost its peptidase activity. In contrast, the epoxide hydrolase activity was intact or even increased as compared to wild type enzyme[31,32]. These results are in line with a role of Glu-296 as a general base in the peptidase reaction.

Furthermore, sequence comparisons with aminopeptidase N suggested that Tyr-383 may act as a proton donor in peptidolysis[33,34,35]. Indeed, when this residue was subjected to mutational analysis, a selective abrogation of the aminopeptidase activity was observed[35], supporting a catalytic role for Tyr-383.

## Evidence for a Carbocation Intermediate in the Epoxide Hydrolase Reaction

Further investigation of the catalytic properties of mutants in position 383 revealed the formation of large quantities of a novel metabolite of $LTA_4$, structurally identified as 5*S*,6*S*-dihydroxy-7,9-*trans*-11,14-*cis*-eicosatetraenoic acid (5*S*,6*S*-DHETE) in addition to the expected $LTB_4$[36]. Analysis of the stereochemistry of the vicinal diol and the positional specificity for incorporation of $H_2{}^{18}O$ revealed that the epoxide hydrolysis must occur according to an $S_N1$ reaction, which involves a carbocation intermediate. Inasmuch as the mutants also produced $LTB_4$, it seems likely that enzymatic hydrolysis of $LTA_4$ into $LTB_4$ follows the same mechanism.

## Glu-271 Plays Specific Roles in Each of the Epoxide Hydrolase and Peptidase Reactions

In the crystal structure of $LTA_4$ hydrolase, Glu-271 was found to be located in the immediate vicinity of the prosthetic zinc and the catalytic residues Glu-296 and Tyr-383[37]. Glu-271 is a component of a GXMEN motif, which is conserved among members of the M1 family of metallopeptidases and proposed to play a role in peptide binding[22]. To detail the role of Glu-

271, we carried out a mutational analysis of all residues within the GXMEN motif[38]. All site-specific mutants retained their catalytic function with the exception of mutants of Glu-271, which had lost not only the peptidase activity but also the epoxide hydrolase activity. Furthermore, the crystal structure of the most conservative mutant at position 271 — that is, [E271Q]$LTA_4$ hydrolase — revealed the presence of the catalytic zinc without any significant structural alterations of the active site. Hence, these combined mutational and structural data strongly indicate that Glu-271 is required for both enzyme reactions.

Considering the location of Glu-271 (close to the catalytic zinc), it seems possible that its side-chain carboxylate is close to the epoxide moiety of $LTA_4$ and actually participates in the activation and opening of the oxirane ring. For the aminopeptidase activity, on the other hand, it seems likely that the carboxylate of Glu-271 functions as an anchor for the N-terminal α-amino group of peptide substrates. In this function, Glu-271 will be important for the exopeptidase specificity of the enzyme and also contribute to substrate alignment, which in turn will influence the formation of a transition-state complex and subsequent substrate turnover. This conclusion also agrees well with previous studies with site-directed mutagenesis of other zinc aminopeptidases[39,40]. Hence, Glu-271 is a unique example of a residue that is shared between two catalytic activities, i.e., the epoxide hydrolase and aminopeptidase activity of $LTA_4$ hydrolase. Yet Glu-271 carries out a separate chemistry in each of the two reaction mechanisms. Apparently, the GXMEN sequence qualifies as a consensus motif for an N-terminal recognition site among members of the M1 family of zinc aminopeptidases.

## CRYSTAL STRUCTURE OF $LTA_4$ HYDROLASE

### The Overall Structure of the Protein

Recently, the x-ray crystal structure of $LTA_4$ hydrolase in complex with the competitive inhibitor bestatin was determined at 1.95 Å resolution[37]. The protein molecule is folded into three domains — N-terminal, catalytic, and C-terminal — that are packed in a flat triangular arrangement with approximate dimensions 85 x 65 x 50 $Å^3$. Although the three domains pack closely and make contact with each other, a deep cleft is created between them (Fig. 2).

### The N-Terminal Domain Is Structurally Related to Bacteriochlorofyll *a*

The N-terminal domain (residue 1–209) is composed of one seven-stranded mixed β-sheet, and one four- and one three-stranded antiparallel β-sheet. Strands from the larger β-sheet continue into the two smaller β-sheets that pack on the edges of the same side of the larger sheet so that a kind of envelope is formed (Fig. 3A). The two small β-sheets are turned towards the inside of the whole protein while the larger β-sheet is exposed to solvent and forms a large concave surface area. The N-terminal domain of $LTA_4$ hydrolase shares important structural features with the chlorophyll-containing enzyme bacteriochlorophyll (Bchl) *a*[41]. Thus, 111 Cα positions have equivalent positions in the two proteins despite the absence of any sequence identity (Fig. 3B). The structural similarity with Bchl *a*, a membrane-associated protein involved in light harvesting, suggests that the N-terminal domain of $LTA_4$ hydrolase may recognize lipid structures, possibly required during the functional coupling of $LTA_4$ hydrolase to the leukotriene biosynthetic complex at the nuclear membrane[42,43].

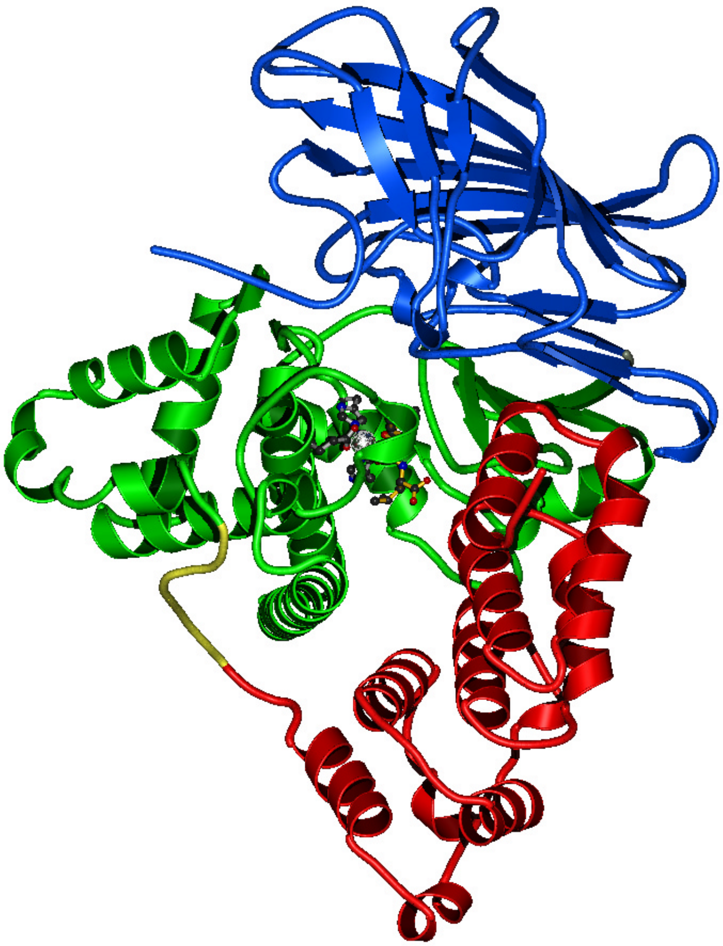

**FIGURE 2.** Overall structure of $LTA_4$ hydrolase. Ribbon diagram of the 3D-structure of $LTA_4$ hydrolase. The N-terminal domain is colored blue, the catalytic domain green, and the C-terminal domain red. A loop (residues 451–460) containing a highly conserved Pro-rich motif and connecting the catalytic and C-terminal domain is shown in yellow. In the central part of the molecule, the catalytic zinc and the three amino acid ligands are shown. The figure was created using MolScript[72] and POV-Ray (http://www.povray.org).

## The Catalytic Domain Contains the Zinc Binding Site and is structurally Similar to Thermolysin

The structure of the catalytic domain (residue 210–450) is surprisingly similar to the structure of thermolysin (Fig. 4)[44]. Although the amino acid sequence is only 7% identical to that of thermolysin (essentially confined to the zinc binding motifs), the structural homology stretches out over the whole domain. Like thermolysin, the catalytic domain consists of two lobes, one mainly α-helical and one mixed α/β-lobe. The α-lobe consists of six major helices interconnected by long loops containing smaller helical segments, while the α/β-lobe has a five-stranded mixed β-sheet lined with three helices on one side. The zinc binding site is found in between the two lobes.

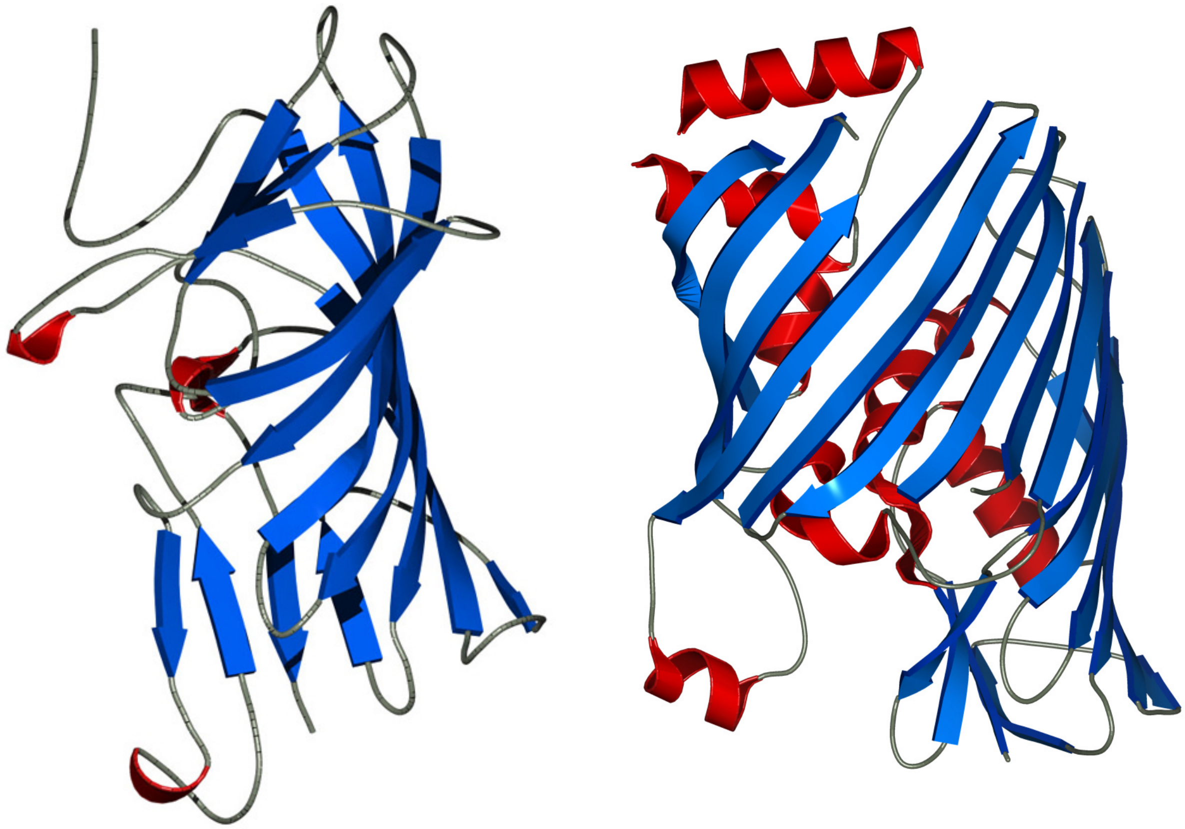

**FIGURE 3.** Structure of the N-terminal domain and comparison with bacteriochlorophyll *a*. Ribbon diagrams of the N-terminal domain of $LTA_4$ hydrolase (A) and bacteriochlorophyll *a* (B). α-Helices are in red, β-strands in blue.

## The C-Terminal Domain Is Reminiscent of Helical Repeat Proteins

The C-terminal domain (residue 464–610) is formed by nine α-helices that form an unusual coil of helices reminiscent of the armadillo repeat or HEAT motif regions, present in proteins such as β-catenin and the nuclear import factor karyopherin α (Fig. 5)[45,46,47]. These folds generally create super-helical structures ideally suited for protein-protein interactions. In $LTA_4$ hydrolase, there are two layers of parallel helices, five in the inner layer and four in the outer, arranged in an antiparallel manner. On top of these layers, longer perpendicular loops containing short helical segments are found. The helices are highly amphipathic in character, with their hydrophobic sides towards the middle of the domain and hydrophilic residues pointing towards the solvent and into the deep cleft in the middle of the whole molecule.

## The Catalytic Zinc Site and a Possible Binding Site for $LTA_4$

The zinc site is located at the bottom of the interdomain cleft. As predicted from our previous work, the metal is bound to the three amino acid ligands, His-295, His-299, and Glu-318. In the structure, the aminopeptidase inhibitor bestatin is also liganding to the $Zn^{2+}$, creating a pentavalent coordination. The inhibitor makes contacts with residues from all three domains. Hydrogen bonds between the protein and all the polar atoms of the inhibitor are made. The

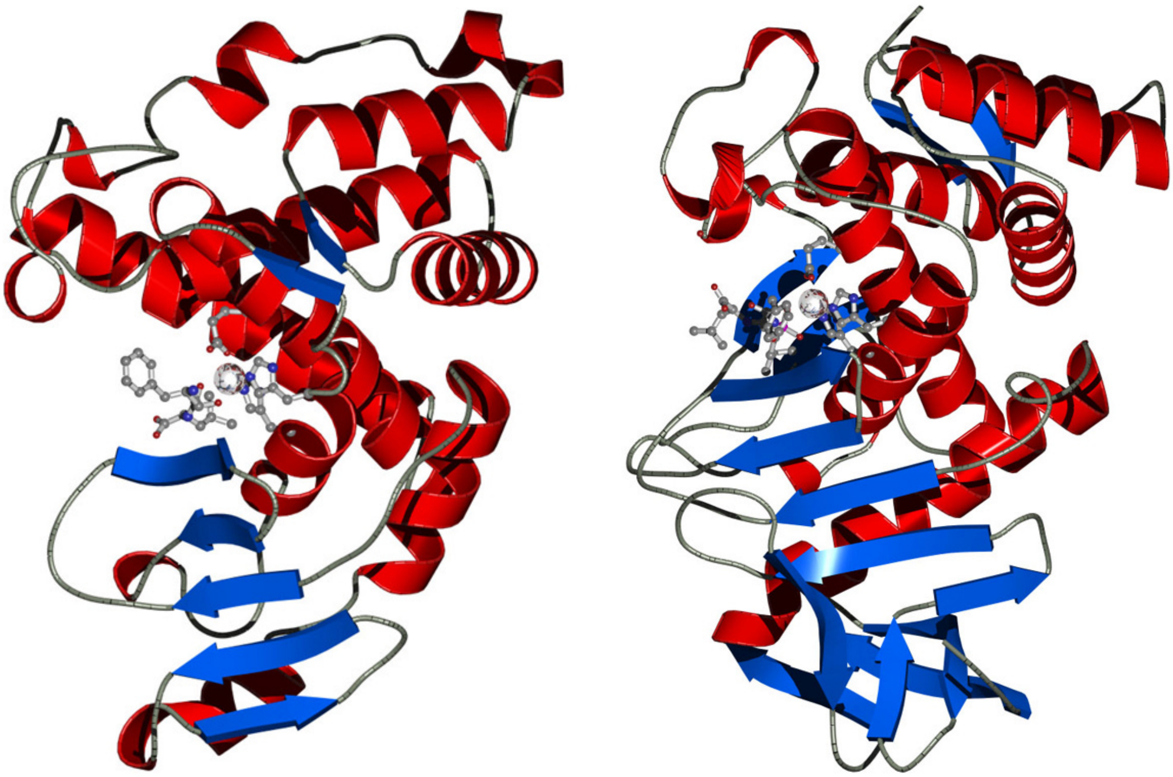

**FIGURE 4.** Structure of the catalytic domain and a comparison with thermolysin. (A) Ribbon diagram of the catalytic domain; α-helices are in red, β-strands are in blue. The three zinc ligands, His-295, His-299, and Glu-318, and the inhibitor bestatin are depicted in ball-and stick representation The zinc ion is shown as a CPK model. (B) Structure of thermolysin in the same orientation as the catalytic domain; α-helices are in red, β-strands are in blue. The three zinc ligands, His-142, His-146, and Glu-166, as well as the inhibitor Cbz-GlyP-(O)-Leu-Leu50, are depicted in ball-and stick representation. The zinc ion is shown as a CPK model.

phenyl group of the inhibitor is bound in an hydrophobic pocket. In the vicinity of the prosthetic zinc, the catalytic residues Glu-296 and Tyr-383 are located at positions that are commensurate with their proposed roles as general base and proton donor in the aminopeptidase reaction. Behind the pocket occupied by the phenyl ring of bestatin there is an L-shaped hydrophobic cavity approximately 6 to 7 Å wide, which stretches 15 Å deeper into the protein. Most of the residues lining the pocket are conserved among $LTA_4$ hydrolases and belong to peptide K21 (Leu-365-Lys-385), thus corroborating our previous conclusion that K21 is a part of the enzyme's active center[26,48]. One patch of the cavity is hydrophilic, with Gln-134, Asp-375, and the hydroxyl of Tyr-267 clustering together.

## PROPOSED CATALYTIC MECHANISMS

Two structural features are important for the biological activity of $LTB_4$: the $\Delta^6$-*cis*-$\Delta^8$-*trans*-$\Delta^{10}$-*trans* double-bond geometry and the S-configuration of the hydroxyl group at C12, both of which must be controlled by $LTA_4$ hydrolase. The epoxide hydrolase reaction, i.e., the conversion of $LTA_4$ into $LTB_4$, is unique in that the stereospecific introduction of a hydroxyl group occurs at a site distant from the epoxide moiety. Very little is known regarding the functional elements and molecular mechanisms of this reaction. Concerning the aminopeptidase activity, it seems to be highly specific for arginyl di- and tripeptides[19]. Unlike the epoxide hydrolase activity, more is

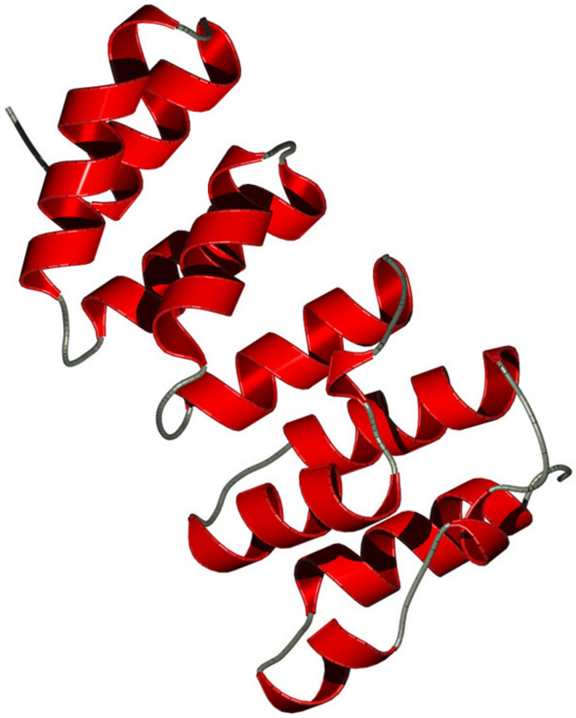

**FIGURE 5.** Structure of the C-terminal domain of $LTA_4$ hydrolase. Ribbon diagram of the double layer of nine α-helices (red) comprising the C-terminal domain.

known about the catalytic mechanisms and which amino acids are involved. Notably, the crystal structure of $LTA_4$ hydrolase provides several important clues as to how the enzyme may execute its sophisticated epoxide hydrolase reaction as well as further evidence for the mechanism of peptide hydrolysis.

## Putative Mechanism for Conversion of $LTA_4$ into $LTB_4$

Assuming that $LTA_4$ binds to the hydrophobic pocket (see the section above entitled "The Catalytic Zinc Site and a Possible Binding Site for $LTA_4$"), the molecule can be modeled into this binding site such that the 5,6-epoxide moiety is near the $Zn^{2+}$ and C-7 to C-20 of the fatty acid backbone sticks deep into the cavity, adopting a bent conformation (Fig. 6A). The C-1 carboxylate could potentially make electrostatic interactions with Arg-563 and/or Lys-565. In this model for $LTA_4$ binding, the catalytic zinc as well as Glu-271 will be proximal to the labile allylic epoxide, suggesting that they polarize a water molecule and promote an acid-induced activation and opening of the epoxide ring (Fig. 6A). A carbocation would be generated whose charge is delocalized over the conjugated triene system (C-6 to C-12), leaving the planar $sp^2$ hybridized C-12 open for nucleophilic attack from either side of the molecule. In this model, the C-12 atom of $LTA_4$ would be in the neighborhood of residues Gln-134, Asp-375, and Tyr-267. These residues could, alone or in concert with each other, direct a water molecule for attack at C-12 and thus control the positional and stereospecific insertion of the 12*R* hydroxyl group in $LTB_4$. Moreover, since there is free rotation between C-6 and C-7 of $LTA_4$, the enzyme may keep this bond in a "pro-*cis*" configuration in the transition state, which would promote the formation of a *cis* double bond from the carbocation intermediate (Fig. 6A). The structure also provides some

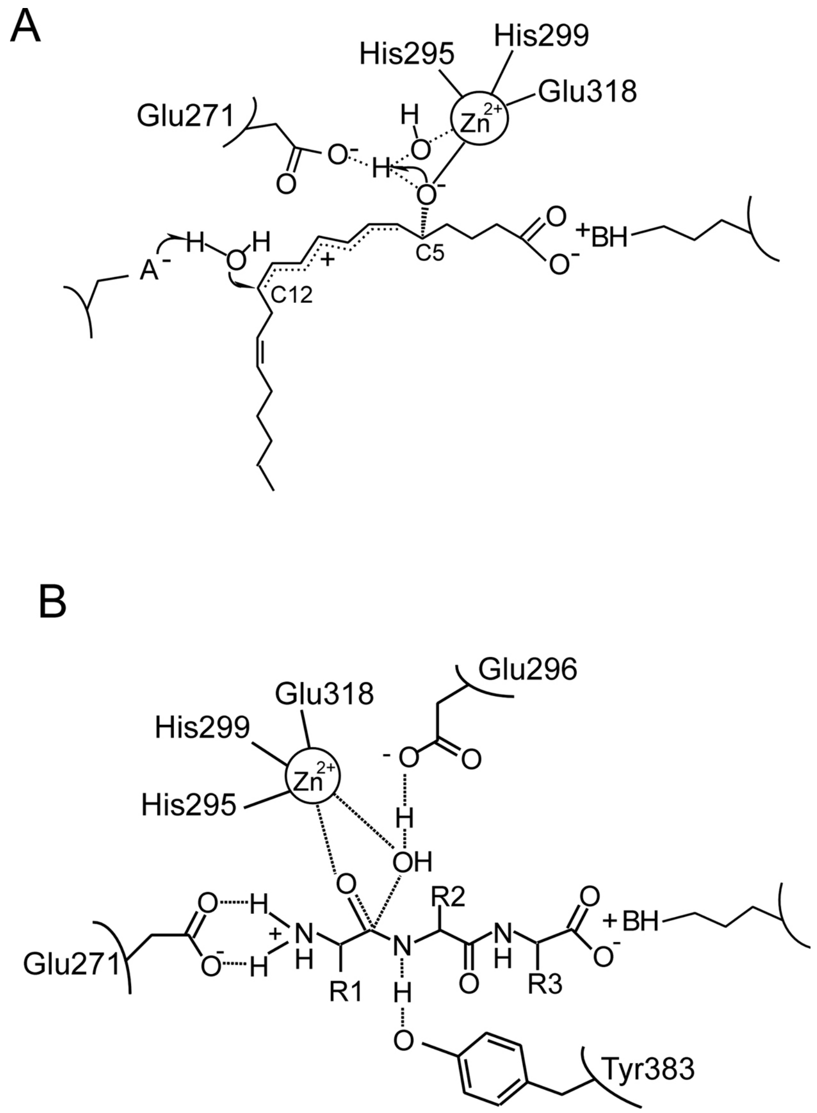

**FIGURE 6.** Putative mechanisms for the epoxide hydrolase and aminopeptidase reactions. (A) In hydrolysis of $LTA_4$, the catalytic zinc, assisted by the carboxylate of Glu-271, polarizes a water molecule to promote an acid induced activation of the epoxide. As a result, the oxirane ring opens up to form a carbocation intermediate according to an $S_N1$ reaction. Water is added at C12 in a stereospecific manner, presumably directed by a polar residue depicted as $A^-$ in the figure. A basic residue, indicated by $BH^+$, probably acts as a carboxylate recognition site. (B) In the aminopeptidase reaction, an activated water molecule bound to the zinc is displaced by a carbonyl group of the incoming tripeptide substrate. The α-aminogroup of the substrate gets attached to Glu-271, acting as an N-terminal recognition site. The water is polarized by the base, Glu-296, and attacks the peptide bond. Simultaneously, a proton is donated from Tyr-383.

information regarding the function of Tyr-378 and Tyr-383. Thus, both residues are hydrogen-bonded to each other and in our model of $LTA_4$ binding they get in close contact with the conjugated triene system just at the angle of the L-shaped binding cavity. In fact, their positions would become ideal for assisting optimal substrate alignment and promoting a specific double bond configuration, in line with what has been proposed from mutational analysis and catalytic properties of [Y378F]- and [Y383Q]$LTA_4$ hydrolases[27,36,48]. Clearly, further studies are required to elucidate and corroborate this model for the mechanism of enzymatic conversion of $LTA_4$ into $LTB_4$. In particular, the role of the hydrophobic, L-shaped cavity for binding of $LTA_4$ needs to be explored.

### Proposed Mechanism for the Aminopeptidase Activity

For the peptidase activity, several structural elements required for substrate binding and catalysis have been identified. Together, they permit us to propose a general base mechanism, in analogy with what has been discussed for thermolysin[29,30]. Since bestatin, the inhibitor present in the structure of $LTA_4$ hydrolase, is a dipeptide mimic, it gives further clues to the binding of a peptide substrate. Thus, a compilation of biochemical, mutagenetic, and structural data leads to the following reaction mechanism[31,35,38]: a water is displaced from the zinc atom by the carbonyl oxygen of the substrate, which in turn gets anchored to the active site via its N-terminal α-amino group binding to Glu-271 (Fig. 6B). The water molecule is simultaneously polarized by the carboxylate of Glu-296 to promote an attack on the carbonyl carbon of the scissile peptide bond. At the same time, a proton is transferred to the nitrogen of the peptide bond by Tyr-383. Since Glu-271 most likely contributes significantly to substrate alignment and the formation of a proper transition state complex, this residue is also important for the overall catalysis.

## MOLECULAR EVOLUTION OF $LTA_4$ HYDROLASE

$LTA_4$ hydrolase is homologous to other zinc aminopeptidases in a variety of species, ranging from mammals to bacteria, in particular those belonging to the M1 family[22]. However, the epoxide hydrolase activity appears to be unique for $LTA_4$ hydrolase and has not been detected with certainty in human homologues such as aminopeptidase B, although conflicting data exist in the literature[49,50]. Furthermore, the epoxide hydrolase activity is not widely spread among homologues of $LTA_4$ hydrolase from lower species. Biosynthesis of $LTB_4$, suggesting the presence of an $LTA_4$ hydrolase, has been observed in several lower vertebrates, including fish and frogs, but not in nonvertebrate species[51,52,53,54], and an $LTA_4$ hydrolase from the African claw toad *Xenopus laevis* has been characterized[55]. Interestingly, this enzyme, which is about 60 to 70% identical to the human enzyme, had an increased catalytic efficiency (tenfold) and the ability to convert $LTA_4$ into two enzymatic products, viz. $LTB_4$ and 6-*trans*-8-*cis*-$LTB_4$. Recently, the first example of a nonvertebrate $LTA_4$ hydrolase with a primitive epoxide hydrolase activity was reported. Thus, an $LTA_4$ hydrolase that is 39% identical (53% similar) to the human enzyme was cloned and characterized from yeast, *Saccharomyces cerevisiae*[56,57]. The *S. cerevisiae* $LTA_4$ hydrolase is a zinc leucyl aminopeptidase with an epoxide hydrolase activity that converts $LTA_4$ into three products, viz. 5*S*,6*S*-dihydroxy-7,9-*trans*-11,14-*cis*-eicosatetraenoic acid (5*S*,6*S*-DHETE), $LTB_4$, and 5*S*,12*R*-dihydroxy-6,10-*trans*-8,14-*cis*-eicosatetraenoic acid ($\Delta^6$-*trans*-$\Delta^8$-*cis*-$LTB_4$). Furthermore, $LTA_4$ can bind in a tight but noncovalent mode to the active site of the *S. cerevisiae* enzyme, which leads to inactivation of the epoxide hydrolase activity and strong activation of the peptidase activity[58]. The yeast enzyme is only slightly, if at all, susceptible to suicide inactivation, and carries a Phe residue at the site corresponding to Tyr-378 in the human protein. Moreover, several point mutations improve the epoxide hydrolase activity and make the catalytic properties more similar to the human enzyme. Apparently, *S. cerevisiae* $LTA_4$ hydrolase is a strong candidate as an early ancestral gene for mammalian $LTA_4$ hydrolases.

An aminopeptidase (AP1) from *Caenorhabditis elegans* was also cloned and characterized[59]. It is 45% identical (63% similar) at the amino acid level to mammalian $LTA_4$ hydrolase and exhibits an arginyl aminopeptidase activity. Notably, despite this high level of sequence identity, the *C. elegans* enzyme fails to hydrolyze $LTA_4$ into $LTB_4$, and no other functional links to $LTA_4$ hydrolase have been reported. Apparently, the degree of sequence identity is not sufficient to denote a gene as an $LTA_4$ hydrolase; this can only be achieved with biochemical methods.

Together, these data indicate that $LTA_4$ hydrolase has developed from an ancestral aminopeptidase, which initially possessed an allosteric lipid-binding site. During evolution, the architecture of this site was gradually changed to become an active site accommodating $LTA_4$. Subsequent optimizations of the structure further improved substrate alignment, and finally allowed efficient catalysis and formation of $LTB_4$. At the same time, the enzyme was penalized by a catalytic restraint imposed by increased susceptibility to suicide inactivation, presumably due to the exchange of a Phe residue for a Tyr, at the active site.

## ORGANIZATION OF THE $LTA_4$ HYDROLASE GENE

The gene structure of human $LTA_4$ hydrolase has also been determined[60]. It is a single copy gene with a size of > 35 kbp. The coding sequence is divided into 19 exons ranging in size from 24 to 312 bp. The human $LTA_4$ hydrolase gene was mapped to chromosome 12q22, using fluorescence *in situ* hybridization. The putative promoter region (approx. 4 kbp) contains a phorbol-ester response element (AP-2) and two xenobiotic-response elements (XRE) but no definitive TATA box. The significance of these putative cis-elements has not been determined.

## $LTA_4$ HYDROLASE–DEFICIENT MICE

Mice deficient in $LTA_4$ hydrolase, and thus the ability to convert $LTA_4$ into $LTB_4$, have been generated by targeted gene disruption[9]. These mice develop normally and are healthy. Analysis of their reactivity against various proinflammatory stimuli revealed that $LTA_4$ hydrolase is required for the formation of $LTB_4$ during an *in vivo* inflammatory reaction. Comparing the phenotype of these mice with that of 5-LO (-/-) mice allowed a delineation of the relative contribution of $LTB_4$ and cys-LTs, respectively, to a specific inflammatory response. Thus, $LTB_4$ is responsible for the characteristic influx of neutrophils, which follows topical application of arachidonic acid and contributes to the vascular changes observed in this inflammatory model. In zymosan-A induced peritonitis, $LTB_4$ modulates only the cellular component of the response, whereas $LTC_4$ appears to be responsible for the plasma protein extravasation. Moreover, $LTA_4$ hydrolase was recently shown to be upregulated in the hearts of angiotensin II–induced hypertensive rats, thus providing further evidence for a role of $LTA_4$ hydrolase in inflammatory reactions *in vivo*[61]. Of note, $LTA_4$ hydrolase (-/-) mice are resistant to the lethal effects of systemic shock induced by PAF, thus identifying $LTB_4$ as a key mediator of this reaction.

## DEVELOPMENT OF ENZYME INHIBITORS

The discovery that $LTA_4$ hydrolase belongs to a family of zinc proteases opened up new possibilities in the search for enzyme inhibitors. Bestatin and captopril are inhibitors of aminopeptidases and angiotensin converting enzyme, respectively, and were also found to be effective inhibitors of $LTA_4$ hydrolase[62]. Furthermore, kelatorphan, a known inhibitor of enkephalin degrading enzymes, is a potent inhibitor of $LTA_4$ hydrolase[63], and a class of ω-[(ω-arylalkyl)aryl]alkanoic acids were reported to inhibit $LTA_4$ hydrolase in the low μM range, one of which was metabolically stable after oral administration to rats[64].

Several laboratories have developed more powerful and selective compounds, based on proposed reaction mechanisms and inhibitor-enzyme interactions for other zinc hydrolases. For example, an α-keto-β-amino ester, a thioamine, and an amino hydroxamic acid were synthesized and found to be effective tight-binding inhibitors with $IC_{50}$ values in the low μM to nM range[65,66,67]. These three compounds were also potent and selective inhibitors of $LTB_4$ biosynthesis in intact human leukocytes.

A series of inhibitors of $LTA_4$ hydrolase have been developed by Searle, e.g., SC-57461, *N*-methyl-*N*-[3-[4-(phenylmethyl)-phenoxy]propyl]-β-alanine, which blocks ionophore-induced $LTB_4$ production in human whole blood with an $IC_{50}$ of 49 nM[68,69,70] and also derivatives of SC-22716, in particular 2-[4-[4-[2-(1-pyrrolidinyl)ethoxy]phenoxy]phenyl]-oxazole[71]. Both compounds are orally active and the former structure has also shown promising results in an animal model of colitis[68]. The structure of $LTA_4$ hydrolase will certainly become a powerful tool to generate better inhibitors by structure-based drug design.

## ACKNOWLEDGMENTS

This work was financially supported by the Swedish Medical Research Council (O3X-10350), the Swedish Natural Sciences Research Council, the European Union (QLG1-CT-2001-01521), the Foundation for Strategic Research, and Konung Gustav V:s 80-Årsfond.
